# Comparative Analysis of Perioperative Analgesia Methods in Thoracic Surgery: A Literature Systemic Review

**DOI:** 10.3390/jcm14072484

**Published:** 2025-04-05

**Authors:** Fahim Kanani, Rijini Nugzar, Mordechai Shimonov, Firas Abu Akar

**Affiliations:** 1 Department of Surgery, The Edith Wolfson Medical Center, Affiliated to the Sackler Faculty of Medicine, Tel Aviv University, Tel Aviv 6997801, Israel; kanani.fahim@gmail.com (F.K.); shimonov.m@wmc.gov.il (M.S.); 2 Department of Anaesthesia, The Edith Wolfson Medical Center, Affiliated to the Sackler Faculty of Medicine, Tel Aviv University, Tel Aviv 6997801, Israel; rijini.nugzar@wmc.gov.il; 3 Department of Thoracic Surgery, The Edith Wolfson Medical Center, Affiliated to the Sackler Faculty of Medicine, Tel Aviv University, Tel Aviv 6997801, Israel

**Keywords:** thoracic surgery, pain management, paravertebral block, intercostal nerve block, epidural analgesia, erector spinae plane block, opioid consumption, postoperative complications

## Abstract

**Background/Objectives:** Effective pain management following thoracic surgery remains challenging yet crucial for optimal patient outcomes. This literature review compares the efficacy, safety, and clinical outcomes of different perioperative analgesia methods in thoracic surgery patients, focusing on paravertebral block (PVB), intercostal nerve block (ICNB), epidural analgesia (EPI), erector spinae plane block (ESPB), and patient-controlled analgesia (PCA). **Methods**: A systematic search was conducted across medical databases, yielding ten relevant randomized controlled trials and meta-analyses. **Results**: The evidence indicates that paravertebral block provides superior pain control with lower opioid requirements, fewer adverse events, and higher patient satisfaction compared to other methods. While epidural analgesia offers pain control comparable to PVB, it is associated with higher technical failure rates and side effects, including urinary retention, nausea/vomiting, and hypotension. ICNB and ESPB demonstrate efficacy superior to systemic analgesia but generally inferior to PVB in terms of pain scores and opioid consumption. **Conclusions**: This review highlights the need for individualized approaches to perioperative pain management in thoracic surgery, with paravertebral block emerging as a preferred option due to its favorable efficacy and safety profile.

## 1. Introduction

Thoracic surgical procedures are associated with significant postoperative pain due to surgical tissue trauma, rib spreading, intercostal nerve damage, and chest tube irritation [1,2]. Inadequate pain control contributes to respiratory complications, delayed mobilization, prolonged hospital stays, and the development of chronic post-thoracotomy pain syndrome [3]. While multiple analgesia techniques have been developed to address these challenges, consensus regarding the optimal approach remains elusive [4].

The evolution of thoracic surgical techniques from traditional open thoracotomy to minimally invasive approaches, including video-assisted thoracoscopic surgery (VATS) and robotic-assisted thoracoscopic surgery (RATS), has influenced perioperative pain management strategies [5,6,7]. Despite these advances in surgical techniques, Jain et al. (2023) demonstrated that optimal analgesia remains essential for enhanced recovery and reduced complications regardless of the surgical approach [8].

This literature review aims to evaluate and compare the efficacy, safety, and clinical outcomes of different perioperative analgesia methods in thoracic surgery patients, with a focus on paravertebral block (PVB), intercostal nerve block (ICNB), epidural analgesia (EPI), erector spinae plane block (ESPB), and patient-controlled analgesia (PCA). By analyzing the current evidence, this review seeks to identify the most effective approach for optimal pain management in thoracic surgery patients.

## 2. Materials and Methods

PRISMA Statement

**Figure 1:**
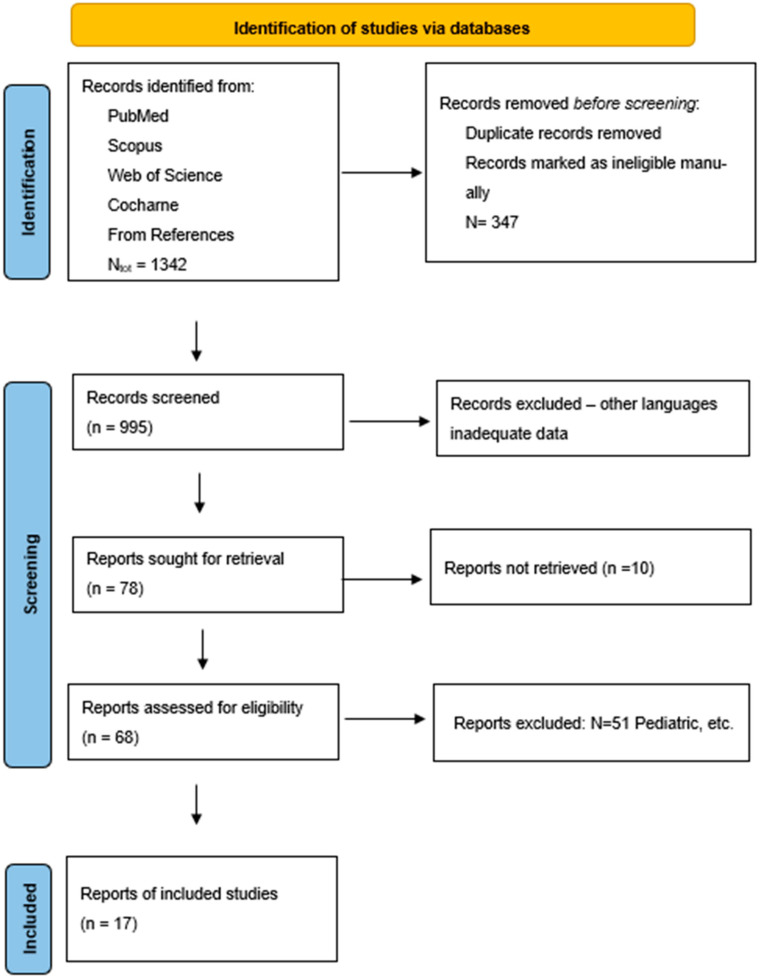
The systemic review included sources from PubMed, Scopus, Cochrane, and Web of Science, as well as sources from references. Source: [9] This element is licensed under CC BY 4.0. To view a copy of this license, visit https://creativecommons.org/licenses/by/4.0/ (accessed on 25 July 2024).

This systematic review was conducted in accordance with the Preferred Reporting Items for Systematic Reviews and Meta-Analyses (PRISMA) 2020 guidelines. The review was not registered in any systematic review database. A comprehensive search strategy was developed to identify relevant studies comparing different perioperative analgesia methods in thoracic surgery with a focus on paravertebral block (PVB), intercostal nerve block (ICNB), epidural analgesia (EPI), erector spinae plane block (ESPB), and patient-controlled analgesia (PCA). The PRISMA flow diagram is presented in Figure 1.

The diagram includes the number of records identified through database searching (n = 1342), records after duplicates removed (n = 995), records screened (n = 995), records excluded (n = 917), full-text articles assessed for eligibility (n = 78), and studies included in qualitative synthesis (n = 17), as well as reasons for the exclusions at the full-text review stage.

A comprehensive search was conducted across major medical databases, including PubMed, Scopus, Web of Science, and the Cochrane Library, searching from inception to February 2025. The search strategy employed the following Boolean combinations: (“thoracic surgery” OR “thoracotomy” OR “VATS” OR “video-assisted thoracoscopic surgery”) AND (“pain management” OR “analgesia” OR “paravertebral block” OR “intercostal block” OR “epidural analgesia” OR “erector spinae plane block” OR “patient-controlled analgesia”). Additional relevant studies were identified through manual searching of reference lists.

The diagram includes the number of records identified through database searching (n = 1342), records after duplicates removed (n = 995), records screened (n = 995), records excluded (n = 917), full-text articles assessed for eligibility (n = 78), and studies included in qualitative synthesis (n = 17), as well as the reasons for exclusions at the full-text review stage. Figure 1.

### 2.1. Inclusion and Exclusion Criteria

Studies were included if they met the following criteria:

1. Adult patients undergoing thoracic surgical procedures;
2. Comparative evaluation of at least one of the specified analgesia interventions;
3. Randomized controlled trial, prospective cohort study, or systematic review/meta-analysis design;
4. Reporting of pain scores, analgesic consumption, complications, or patient satisfaction as outcomes;
5. Published in peer-reviewed journals.

Studies were excluded if they involved:

- Pediatric populations;
- Non-thoracic procedures;
- Case reports/series;
- Animal studies;
- An exclusive focus on chronic post-thoracotomy pain.

### 2.2. Data Extraction and Quality Assessment

The data extraction focused on study design, sample size, intervention characteristics, primary outcomes (pain scores, opioid consumption), secondary outcomes (adverse events, patient satisfaction, and recovery metrics), and key findings. The methodological quality of the included randomized controlled trials was assessed using the Cochrane Risk of Bias Tool 2.0, evaluating domains of randomization process, deviations from intended interventions, missing outcome data, measurement of outcomes, and selection of reported results. For systematic reviews and meta-analyses, the AMSTAR-2 tool was applied. Two reviewers independently performed quality assessments, with discrepancies resolved through discussion with a third reviewer.

To assess publication bias, we had planned to use funnel plots and Egger’s test for outcomes reported by ≥10 studies. However, due to the limited number of studies for each comparison, formal assessment of publication bias was not feasible. This limitation is acknowledged in our discussion.

## 3. Results

### 3.1. Characteristics of Included Studies

Ten studies met the inclusion criteria: seven randomized controlled trials, two meta-analyses (one combined with a systematic review), and one systematic review. Sample sizes ranged from 24 to 5184 participants across the studies. Most studies compared two or more analgesia techniques, with paravertebral block being the most commonly evaluated intervention (seven studies), followed by intercostal nerve block (five studies) and epidural analgesia (four studies). Please note Table 1. The quality of studies is shown in the details.

### 3.2. Comparative Efficacy of Analgesia Techniques

Paravertebral block (PVB) consistently demonstrated superior pain control compared to intercostal nerve block (ICNB) and erector spinae plane block (ESPB) at 24 h post-surgery. PVB showed either equivalent or better outcomes compared to thoracic epidural analgesia across most studies. Opioid consumption was generally lower with PVB compared to ICNB/ESPB techniques, though results were mixed when compared to epidural approaches. Limited patient satisfaction data favored PVB (74%) over ICNB (44%). Overall, evidence suggests PVB provides optimal pain management with reduced opioid requirements following thoracic procedures(Table 2).

#### 3.2.1. Pain Control

Pain scores served as the primary outcome measure in eight of the ten studies. Chen et al. (2020) demonstrated that paravertebral block provided significantly lower visual analog scale (VAS) pain scores compared to erector spinae plane block at 0, 2, 4, and 8 h post-operation, and lower scores than intercostal nerve block at 8 h [7]. Similarly, Hutchins et al. (2017) reported lower maximum pain scores with continuous paravertebral catheters compared to single-shot intercostal blocks (3.65 vs. 6.44, *p* < 0.001) [13].

Fortier et al. (2012) found that thoracic paravertebral block resulted in significantly lower VAS pain scores compared to patient-controlled analgesia (*p* < 0.0026) [11]. Turhan et al. (2020) observed that thoracic paravertebral block achieved better pain control than both erector spinae plane block and intercostal nerve block at 24 h post-operation (*p* < 0.017) [17].

**Figure 2:**
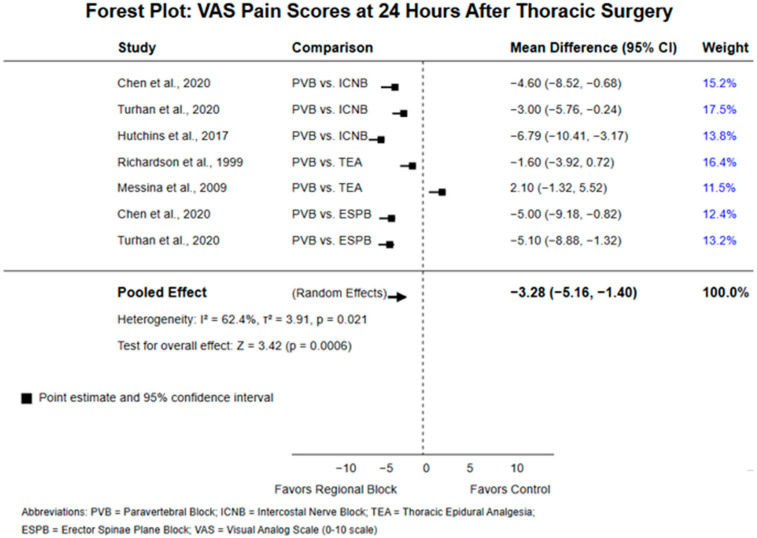
Forest plot—VAS pain scores at 24 h after thoracic surgery [7,13,14,16,17].

When comparing paravertebral block to epidural analgesia, Ding et al.’s (2014) meta-analysis of 18 trials found no significant difference in pain scores at multiple time points (4–8 h, 24 h, 48 h) (mean difference 0.06; 95% confidence interval: −0.31 to 0.42; *p* = 0.77) [10]. Richardson et al. (1999) reported lower pain scores in the paravertebral group compared to the epidural group, though the exact values were not specified [16]. A comprehensive forest chart is shown in Figure 2.

#### 3.2.2. Opioid Consumption

Opioid requirements were reported in nine of the ten studies, providing a quantitative measure of analgesic efficacy. Chen et al. (2020) found significantly lower 24 h morphine consumption with paravertebral block (10.5 mg) compared to intercostal nerve block (18 mg) and erector spinae plane block (22 mg) [7]. Hutchins et al. (2017) reported substantially reduced opioid use with paravertebral catheters (14.39 mg) versus intercostal blocks (30.50 mg) during the 24–48 h postoperative period (*p* = 0.046) [13].

Fortier et al. (2012) demonstrated lower morphine consumption at 24 h with thoracic paravertebral block compared to patient-controlled analgesia (*p* = 0.0036) [11]. In contrast, Messina et al. (2009) found that epidural analgesia required less morphine (9 mg) than paravertebral block (36 mg) (*p* = 0.003) [14].

The meta-analysis by Ding et al. (2014) showed no significant difference in morphine consumption during the first 24 h between paravertebral block and epidural analgesia (mean difference 1.11; 95% confidence interval: −2.20 to 4.41; *p* = 0.51) [10].

#### 3.2.3. Patient Satisfaction

Only one study, Hutchins et al. (2017), reported on patient satisfaction, finding significantly higher satisfaction rates with paravertebral catheters (74%) compared to intercostal blocks (44%) (*p* = 0.036) [13]. This limited reporting highlights a notable gap in the literature regarding patient-centered outcomes in thoracic analgesia research.

### 3.3. Safety and Complications

Paravertebral blocks (PVB) demonstrated superior safety profiles compared to epidural techniques, with significantly lower rates of urinary retention, nausea/vomiting, and hypotension. PVB was associated with fewer failed blocks and better respiratory outcomes including higher oxygen saturations. While epidural techniques showed better spirometry values in one study, PVB generally resulted in reduced respiratory morbidity. Limited recovery data suggested shorter hospital stays with PVB compared to interpleural techniques. Overall, evidence indicates PVB offers comparable efficacy with fewer systemic complications, though many studies lacked comprehensive reporting of adverse events. Please note details of safety in Table 3.

#### 3.3.1. Technical Complications

Ding et al.’s (2014) meta-analysis reported lower failed block rates with paravertebral block compared to epidural analgesia (odds ratio 0.51; *p* = 0.01) [10]. Other studies did not specifically report on technical complications, representing another gap in the comprehensive evaluation of these techniques.

#### 3.3.2. Adverse Events

Six studies reported data on adverse events associated with different analgesic methods. Ding et al. (2014) found that paravertebral block was associated with significantly lower rates of urinary retention (odds ratio 0.21; *p* < 0.0001), nausea/vomiting (odds ratio 0.49; *p* = 0.01), and hypotension (odds ratio 0.11; *p* < 0.00001), compared to epidural analgesia [10]. Similarly, Richardson et al. (1999) observed more side effects, including nausea, vomiting, and hypotension, in patients receiving epidural analgesia, compared to paravertebral block [16].

Joshi et al. (2008) reported a reduced incidence of hypotension with paravertebral block, compared to epidural analgesia [1]. Richardson et al. (1995) noted temporary confusion in five patients in the interpleural analgesia group, compared to none in the paravertebral group (*p* = 0.02) [15].

#### 3.3.3. Recovery Metrics

Five studies reported on recovery-related outcomes. Joshi et al. (2008) found reduced pulmonary complications with paravertebral block, compared to systemic analgesia [1]. Richardson et al. (1999) observed higher oxygen saturations and less respiratory morbidity with paravertebral block, compared to epidural analgesia [16]. Richardson et al. (1995) reported lower postoperative pulmonary complication rates and shorter hospital stays with paravertebral block, compared to interpleural analgesia [15].

Messina et al. (2009) found better spirometer values at 72 h with epidural analgesia, compared to paravertebral block (*p* = 0.03) [14]. Ding et al. (2014) reported no significant difference in pulmonary complications between paravertebral block and epidural analgesia (odds ratio 0.51; *p* = 0.09) [10].

## 4. Discussion

### 4.1. Comparison of Analgesia Techniques

The evidence from this literature review suggests that regional analgesia techniques generally provide superior pain control, compared to systemic approaches, in thoracic surgery patients. Among the regional techniques, paravertebral block emerges as a particularly effective option, demonstrating its advantages in multiple studies.

#### 4.1.1. Paravertebral Block vs. Intercostal Nerve Block

Multiple studies consistently demonstrated the superiority of paravertebral block over intercostal nerve block in terms of pain control, opioid consumption, and patient satisfaction [1,7,8]. Chen et al. (2020) found that paravertebral block provided more sustained analgesia, with benefits extending to 8 h post-operation, compared to intercostal nerve block [7]. Hutchins et al. (2017) highlighted the advantage of continuous paravertebral catheter techniques, which allowed for prolonged analgesia during the critical 24–48 h postoperative period [13].

The enhanced efficacy of paravertebral block may be attributed to its mechanism of action, affecting multiple contiguous dermatomes through a single injection and blocking both somatic and sympathetic nerves [15]. Additionally, the potential for catheter placement allows for continuous infusion, addressing both immediate and delayed postoperative pain [23].

#### 4.1.2. Paravertebral Block vs. Epidural Analgesia

The comparison between paravertebral block and epidural analgesia revealed interesting nuances. While Ding et al.’s (2014) meta-analysis found analgesic efficacy to be comparable between the two techniques [10], Richardson et al. (1999) reported better pain control with paravertebral block [16]. This discrepancy may reflect differences in study populations, surgical procedures, or technical execution.

The most striking differences emerged in the safety profile, with paravertebral block demonstrating significantly lower rates of urinary retention, nausea/vomiting, and hypotension, compared to epidural analgesia [10,17]. Furthermore, the lower technical failure rate of paravertebral block represents an important practical advantage in clinical settings [17].

The unilateral nature of paravertebral block, targeting only the operated side, may contribute to its favorable hemodynamic profile compared to the bilateral sympathectomy associated with epidural analgesia [18]. This characteristic makes paravertebral block particularly valuable in patients with cardiopulmonary comorbidities or those at risk for hypotension [24].

#### 4.1.3. Regional vs. Systemic Analgesia

The superiority of regional techniques over systemic analgesia was consistently observed across studies. Fortier et al. (2012) demonstrated better pain control and reduced opioid requirements with thoracic paravertebral block, compared to patient-controlled analgesia [11]. Guerra-Londono et al. (2021) found that even intercostal nerve block, while inferior to other regional techniques, was superior to systemic analgesia [12].

The advantages of regional analgesia extend beyond pain control. Joshi et al. (2008) reported reduced pulmonary complications with paravertebral block, compared to systemic analgesia [1], highlighting the potential for regional techniques to positively influence clinically relevant outcomes beyond subjective pain experiences.

#### 4.1.4. Emerging Techniques

The evaluation of newer techniques such as erector spinae plane block revealed promising but limited evidence. Chen et al. (2020) and Turhan et al. (2020) both found erector spinae plane block to be less effective than paravertebral block for postoperative analgesia in thoracic surgery [7,17]. However, the technical simplicity and potentially lower risk profile of erector spinae plane block warrant further investigation [12,25].

### 4.2. Clinical Implications

The findings of this review have several important clinical implications for perioperative pain management in thoracic surgery:

1. Paravertebral block as a first-line option: The consistent evidence supporting the efficacy and safety of paravertebral block suggests it should be considered a first-line analgesic approach for thoracic surgery patients.
2. Patient-specific considerations: While paravertebral block demonstrates overall favorable outcomes, individual patient factors such as coagulation status, anatomy, and comorbidities should guide technique selection.
3. Continuous catheter techniques: The extended analgesic benefits observed with continuous paravertebral catheters highlight the importance of addressing pain beyond the immediate postoperative period.
4. Multimodal approaches: The variability in outcomes across studies suggests that multimodal analgesia, combining regional techniques with appropriate systemic agents, may offer the most comprehensive pain management strategy.
5. Technical expertise: The lower failure rates associated with paravertebral block compared to epidural analgesia underscore the importance of procedural competence and institutional experience.

**Figure 3:**
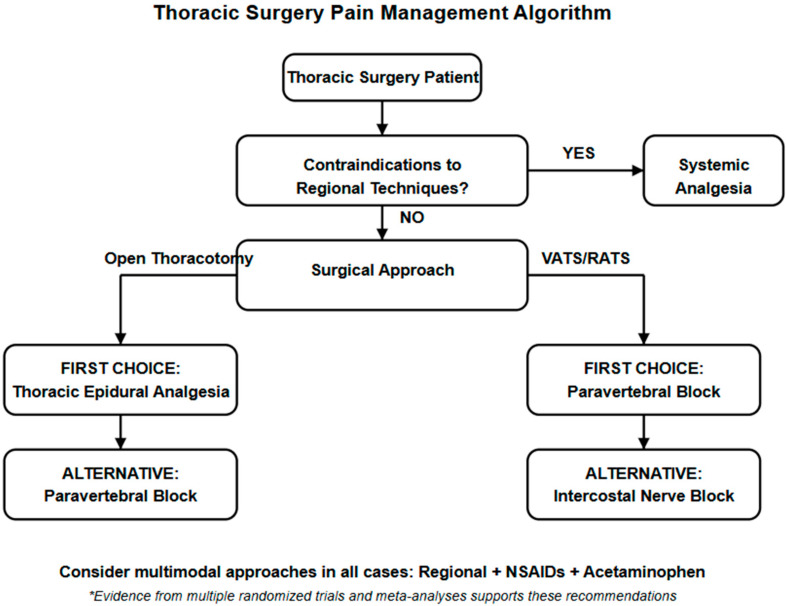
Thoracic surgery pain management algorithm. * based on the Evidence from multiple randomized trials and meta-analyses [1,2,3,4,5,6,7,8,9,10,11,12,13,14,15,16,17,18,20,23,24,25].

A suggestion for a comprehensive thoracic surgery pain management algorithm is shown in Figure 3.

### 4.3. Research Gaps and Future Directions

This review identified several notable gaps in the current literature:

1. Limited reporting of patient satisfaction: Only one study explicitly reported patient satisfaction outcomes, highlighting the need for more patient-centered assessments in future research.
2. Inconsistent time points for pain assessment: The variability in pain measurement timing across studies complicates direct comparisons and meta-analyses.
3. Minimal data on oral analgesics: None of the identified studies included comprehensive evaluations of oral analgesic regimens, representing a significant gap in the literature.
4. Limited long-term follow-up: Most studies focused on immediate postoperative outcomes, with little attention to potential long-term effects such as chronic post-thoracotomy pain.

Future research should address these gaps through well-designed randomized controlled trials with standardized outcome measures, consistent assessment time points, and longer follow-up periods. Additionally, studies examining the cost-effectiveness of different techniques and their impact on rehabilitation and quality of life would provide valuable insights for clinical decision-making.

## 5. Conclusions

This literature review demonstrates that regional analgesia techniques, particularly paravertebral block, provide superior pain control with fewer side effects, compared to systemic analgesia, in thoracic surgery patients. A paravertebral block offers analgesic efficacy comparable to epidural analgesia, but with a more favorable safety profile, including lower rates of urinary retention, nausea/vomiting, and hypotension. The evidence suggests that paravertebral block should be considered a preferred approach for perioperative pain management in thoracic surgery, though individual patient factors and institutional expertise should inform the final selection of technique.

While substantial evidence supports current practice, gaps in the literature highlight opportunities for future research to better define optimal analgesic strategies, particularly regarding patient satisfaction, oral analgesic regimens, and long-term outcomes. As thoracic surgical techniques continue to evolve, parallel advances in pain management approaches will be essential to optimize patient recovery and satisfaction.

## Figures and Tables

**Table 1:** Characteristics of included studies with quality assessment.

Study	Study Design	Sample Size	Intervention Types	Primary Outcomes	Quality Assessment
Chen et al., 2020 [7]	Randomized controlled trial	72	Paravertebral block (PVB), Intercostal nerve block (ICNB), Erector spinae plane block (ESPB)	Cumulative morphine consumption at 24 h postoperatively	Low risk of bias; adequate randomization and allocation concealment; double-blinded design
Ding et al., 2014 [10]	Meta-analysis	777 (across 18 trials)	Paravertebral blockade (PVB), Epidural analgesia (EPI)	Pain scores at 4–8 h, 24 h, 48 h postoperatively; Morphine usage during first 24 h	Moderate quality (AMSTAR-2); comprehensive search strategy; appropriate risk of bias assessment
Fortier et al., 2012 [11]	Randomized controlled trial	140	Systemic analgesia (patient-controlled analgesia, PCA), Continuous wound catheter (CWC), Thoracic paravertebral block (TPVB)	Pain score at rest as assessed by Visual Analog Scale (VAS)	Some concerns; adequate randomization but unclear blinding of outcome assessment
Guerra-Londono et al., 2021 [12]	Systematic review and meta-analysis	5184 (across 66 studies)	Intercostal nerve block (ICNB)	Analgesic benefit during first 24 h after thoracic surgery	High quality (AMSTAR-2); comprehensive search; appropriate statistical methods; publication bias assessment
Hutchins et al., 2017 [13]	Randomized controlled trial	48	Ultrasound-guided paravertebral (PV) catheter, Single-shot intercostal blocks (ICB)	Maximum pain score during 24 to 48 h post-surgery	Some concerns; adequate randomization but incomplete blinding
Joshi et al., 2008 [1]	Systematic review	Not specified	Thoracic epidural, Paravertebral, Intrathecal, Intercostal, and Interpleural analgesic techniques	Postoperative pain, Analgesic use, and Complications	Low quality (AMSTAR-2); limited search strategy; inadequate risk of bias assessment
Messina et al., 2009 [14]	Randomized controlled trial	24	Paravertebral block, Epidural analgesia	Postoperative pain and Respiratory function	High risk of bias; small sample size; inadequate blinding
Richardson et al., 1995 [15]	Randomized controlled trial	53 (45 analyzed)	Interpleural analgesia, Paravertebral analgesia	Visual analogue pain scores (VAS), Patient-controlled morphine (PCM) requirements	Some concerns; adequate randomization but incomplete blinding; high attrition rate
Richardson et al., 1999 [16]	Randomized controlled trial	100	Thoracic epidural bupivacaine, Thoracic paravertebral bupivacaine	Visual analogue pain scores at rest and on coughing	Low risk of bias; adequate randomization and allocation concealment; appropriate blinding
Turhan et al., 2020 [17]	Randomized controlled trial	106	Erector spinae plane block (ESPB), Thoracic paravertebral block (TPVB), Intercostal nerve block (ICNB)	Dynamic visual analog scale in the first hour postoperatively	Low risk of bias; adequate randomization and allocation concealment; appropriate blinding
Jain et al., 2023 [8]	Systematic review	58 studies included (after screening 480 records)	Enhanced Recovery After Surgery (ERAS) protocols with preoperative, intraoperative, and postoperative components	Recovery time; Hospital length of stay; Complication rates; Patient satisfaction	High quality; comprehensive search strategy using PRISMA methodology; appropriate inclusion criteria; thorough review of evidence
Davies et al., 2006 [18]	Meta-analysis	520 (across 10 trials)	Paravertebral blockade, Thoracic epidural analgesia	Pain scores; Analgesia-related side effects; Pulmonary complications	Moderate quality (AMSTAR-2); comprehensive search; appropriate statistical methods
Kotemane et al., 2010 [4]	Systematic review	Not specified	Thoracic epidural, Paravertebral, Intercostal, and other analgesic techniques	Technique prevalence and effectiveness in UK practice	Low quality (AMSTAR-2); limited methodology description; no formal quality assessment
Yeung et al., 2016 [19]	Meta-analysis	1120 (across 14 trials)	Paravertebral block, Thoracic epidural	Pain scores; Analgesia-related complications; Pulmonary outcomes	High quality (AMSTAR-2); comprehensive search; rigorous risk of bias assessment; publication bias evaluation
Pintaric et al., 2011 [20]	Randomized controlled trial	32	Continuous thoracic epidural, Continuous paravertebral block	Hemodynamic stability; Analgesia efficacy; Stress hormone levels	Some concerns; adequate randomization but incomplete blinding of outcomes
Kaiser et al., 1998 [21]	Randomized controlled trial	30	Extrapleural analgesia, Epidural analgesia	Pain scores; Pulmonary function; Patient satisfaction	Some concerns; adequate randomization but unclear allocation concealment
Bimston et al., 1999 [22]	Randomized controlled trial	50	Continuous paravertebral infusion, Thoracic epidural analgesia	Pain control; Respiratory function; Side effects	Some concerns; adequate randomization but incomplete outcome data

Quality Assessment based on Cochrane Risk of Bias Tool 2.0 for randomized controlled trials and AMSTAR-2 for systematic reviews and meta-analyses.

**Table 2:** Comparative pain control effectiveness.

Study	Intervention	Pain Scores (24 h)	Opioid Consumption	Patient Satisfaction
Chen et al., 2020 [7]	PVB, ICNB, ESPB	VAS scores lower in PVB vs. ESPB at 0, 2, 4, 8 h; lower in PVB vs. ICNB at 8 h	PVB: 10.5 mg, ICNB: 18 mg, ESPB: 22 mg (24 h)	No mention found
Ding et al., 2014 [10]	PVB vs. EPI	No significant difference (mean difference 0.06; 95% CI: −0.31 to 0.42; *p* = 0.77)	No significant difference (mean difference 1.11; 95% CI: −2.20 to 4.41; *p* = 0.51)	No mention found
Fortier et al., 2012 [11]	TPVB, CWC, PCA	VAS scores lower in TPVB vs. PCA (*p* < 0.0026)	Lower in TPVB vs. PCA at 24 h (*p* = 0.0036)	No mention found
Guerra-Londono et al., 2021 [12]	ICNB	Superior to systemic analgesia, non-inferior to other techniques	Reduction in consumption, but inferior to thoracic epidural and PVB	No mention found
Hutchins et al., 2017 [13]	PV Catheter vs. ICB	Lower in PV catheter group (3.65 vs. 6.44, *p* < 0.001)	PV: 14.39 mg vs. ICB: 30.50 mg (24–48 h, *p* = 0.046)	PV: 74% vs. ICB: 44% (*p* = 0.036)
Joshi et al., 2008 [1]	Various Regional Techniques	Continuous PVB as effective as thoracic epidural	No mention found	No mention found
Messina et al., 2009 [14]	PVB vs. Epidural	No significant difference	Epidural: 9 mg vs. PVB: 36 mg (*p* = 0.003)	No mention found
Richardson et al., 1995 [15]	Interpleural vs. PVB	Similar VAS scores	Similar PCM use	No mention found
Richardson et al., 1999 [16]	PVB vs. Epidural	Lower in PVB group	Lower in PVB group	No mention found
Turhan et al., 2020 [17]	ESPB, TPVB, ICNB	Lower in TPVB vs. ESPB and ICNB at 24 h (*p* < 0.017)	Similar in TPVB and ICNB, lower than ESPB (*p* < 0.017)	No mention found

**Table 3:** Safety and complications.

Study	Intervention	Technical Complications	Adverse Events	Recovery Metrics
Chen et al., 2020 [7]	PVB, ICNB, ESPB	No mention found	No mention found	No mention found
Ding et al., 2014 [10]	PVB vs. EPI	Lower failed block rates with PVB (odds ratio 0.51; *p* = 0.01)	Lower urinary retention (OR 0.21; *p* < 0.0001), nausea/vomiting (OR 0.49; *p* = 0.01), hypotension (OR 0.11; *p* < 0.00001) with PVB	No significant difference in pulmonary complications (OR 0.51; *p* = 0.09)
Fortier et al., 2012 [11]	TPVB, CWC, PCA	No mention found	No signs of toxicity or local complications observed	No mention found
Guerra-Londono et al., 2021 [12]	ICNB	No mention found	No mention found	No mention found
Hutchins et al., 2017 [13]	PV Catheter vs. ICB	No mention found	No mention found	No mention found
Joshi et al., 2008 [1]	Various Regional Techniques	No mention found	Reduced incidence of hypotension with PVB vs. epidural	Reduced pulmonary complications with PVB vs. systemic analgesia
Messina et al., 2009 [14]	PVB vs. Epidural	No mention found	No mention found	Better spirometer values at 72 h with epidural (*p* = 0.03)
Richardson et al., 1995 [15]	Interpleural vs. PVB	No mention found	Five patients with temporary confusion in interpleural group (*p* = 0.02)	Lower PORM and shorter hospital stay with PVB
Richardson et al., 1999 [16]	PVB vs. Epidural	No mention found	More side effects (nausea, vomiting, hypotension) in epidural group	Higher oxygen saturations and less respiratory morbidity with PVB
Turhan et al., 2020 [17]	ESPB, TPVB, ICNB	No mention found	Similar side effects among groups	No mention found

## Data Availability

No new data were created or analyzed in this study. Data sharing is not applicable to this article as no datasets were generated or analyzed during the current study. The systematic review draws on previously published studies that are cited throughout the manuscript.

## Abbreviations

PVB	Paravertebral block
TPVB	Thoracic paravertebral block
ICNB	Intercostal nerve block
ICB	Intercostal block
EPI	Epidural analgesia
ESPB	Erector spinae plane block
PCA	Patient-controlled analgesia
CWC	Continuous wound catheter
VAS	Visual analog scale
NRS	Numeric rating scale
VATS	Video-assisted thoracoscopic surgery
TEA	Thoracic epidural analgesia
PORP	Postoperative respiratory parameters
RCT	Randomized controlled trial
OR	Odds ratio
CI	Confidence interval
PONV	Postoperative nausea and vomiting
MD	Mean difference
CPTP	Chronic post-thoracotomy pain
